# Utility of MicroRNAs and siRNAs in Cervical Carcinogenesis

**DOI:** 10.1155/2015/374924

**Published:** 2015-03-22

**Authors:** Sacnite del Mar Díaz-González, Jessica Deas, Odelia Benítez-Boijseauneau, Claudia Gómez-Cerón, Victor Hugo Bermúdez-Morales, Mauricio Rodríguez-Dorantes, Carlos Pérez-Plasencia, Oscar Peralta-Zaragoza

**Affiliations:** ^1^Direction of Chronic Infections and Cancer, Research Center in Infection Diseases, National Institute of Public Health, Avenida Universidad No. 655, Cerrada los Pinos y Caminera, Colonia Santa María Ahuacatitlán, 62100 Cuernavaca, MOR, Mexico; ^2^National Institute of Genomic Medicine, Periférico Sur No. 4809, Colonia Arenal Tepepan, Delegación Tlalpan, 14610 Mexico City, DF, Mexico; ^3^Oncogenomics Laboratory, National Cancer Institute of Mexico, Tlalpan, Avenida San Fernando No. 22, Colonia Sección XVI, Delegación Tlalpan, 14080 Mexico City, DF, Mexico; ^4^Biomedicine Unit, FES-Iztacala UNAM, Avenida De los Barrios s/n. Colonia Los Reyes Iztacala, 54090 Tlalnepantla de Baz, MEX, Mexico

## Abstract

MicroRNAs and siRNAs belong to a family of small noncoding RNAs which bind through partial sequence complementarity to 3′-UTR regions of mRNA from target genes, resulting in the regulation of gene expression. MicroRNAs have become an attractive target for genetic and pharmacological modulation due to the critical function of their target proteins in several signaling pathways, and their expression profiles have been found to be altered in various cancers. A promising technology platform for selective silencing of cell and/or viral gene expression using siRNAs is currently in development. Cervical cancer is the most common cancer in women in the developing world and sexually transmitted infection with HPV is the cause of this malignancy. Therefore, a cascade of abnormal events is induced during cervical carcinogenesis, including the induction of genomic instability, reprogramming of cellular metabolic pathways, deregulation of cell proliferation, inhibition of apoptotic mechanisms, disruption of cell cycle control mechanisms, and alteration of gene expression. Thus, in the present review article, we highlight new research on microRNA expression profiles which may be utilized as biomarkers for cervical cancer. Furthermore, we discuss selective silencing of HPV E6 and E7 with siRNAs which represents a potential gene therapy strategy against cervical cancer.

## 1. Introduction

Rapid advances in the study of microRNA expression profiles and siRNAs for silencing gene expression have led to many ongoing efforts to exploit these molecules as biomarkers and therapeutic agents, respectively, in the treatment of several cancers. A key feature of microRNAs and siRNAs is that they are not translated into proteins but rather function in the regulation of gene expression. The study of microRNAs which negatively regulate gene expression by either translational repression or target mRNA degradation, as well as siRNAs which are involved in the biological process of RNA interference, will have greater impact once these molecules are characterized in context of their function and their impact in human health and disease. Considerable progress has occurred into the area of microRNAs and siRNAs in recent years. The new knowledge has contributed to an improved understanding of the mechanism of microRNAs biogenesis and an emerging consensus about the function of microRNAs and their targets in several species including humans. One of the most successful approaches utilizes microRNAs as biomarkers in several diseases. The siRNAs, on the other hand, represent a fast, cost-effective, and relatively simple tool for inducing downregulation of virtually any gene sequence in many species. Therefore, siRNA-based drugs may be the next generation of biochemical compounds because they are highly gene-specific due to nucleotide complementarity and have less challenging pharmacodynamics because siRNAs are biologic molecules.

Cervical cancer is the second most common type of cancer in women in worldwide, with about 500,000 new cases diagnosed and 270,000 women dying each year from this neoplasia [1]. The main causative agent is persistent infection with high-risk human papillomavirus (HPV) and the process of tumorigenesis is associated with well-defined clinical stages, each with unique features [1]. HPV E6 and E7 oncoproteins deregulate cell proliferation and inhibit the apoptotic mechanism mainly by targeting p53 and pRb tumor suppressor proteins, respectively. Additional genetic and epigenetic alterations disrupting cell cycle control are required to immortalize and transform the epithelial host cells [2]. Analysis of global mRNA expression, known as the transcriptome, has demonstrated that aberrant expression of cellular microRNAs has an important role in cervical carcinogenesis [3]. Prior studies have reported deregulation of microRNA expression profiles in various cervical cancer cell lines and tissues compared to normal tissue [3]. In addition, several groups are developing an attractive technology platform to selectively silence gene expression and target HPV E6 and E7 oncogenes, which have significant biological roles in the survival of cervical tumor cells* in vitro* as well as* in vivo*, which represents a potential gene therapy against cervical cancer.

## 2. MicroRNA Biogenesis

In microRNA biogenesis, events begin within the cell nucleus. Primary microRNAs (pri-microRNAs) are processed to generate an intermediate RNA called precursor microRNA (pre-microRNA). Pre-microRNAs are 60 to 110 nucleotides long and form secondary stem-loop-type structures. The processing of pri-microRNAs to generate pre-microRNAs is mediated by an RNase endonuclease type III called Drosha, which hydrolyzes the RNA strands at sites near the base of the stem-loop secondary structure [7–11]. The pre-microRNAs are then exported to the cytoplasm by the Ran guanine nucleotide exchanger (Ran-GTP) and the Exportin-5 receptor [12]. In the next step, the pre-microRNAs are processed by a second cytoplasmic RNase type III called Dicer to produce mature dsRNAs which are 19–25 nucleotides long. These dsRNAs are separated into single strands to generate mature microRNAs [13, 14]. In the next step of microRNA biogenesis, a single microRNA is incorporated into a ribonucleoprotein effector complex known as the RNAi-induced silencing complex (RISC) [15, 16]. The RISC with the mature microRNA guide incorporated identifies the target mRNA via nucleotide base complementarity and either produces endonucleolytic cleavage of the mRNA or induces translation arrest. Several studies have identified the Ago2, Dicer, and protein cofactors as components of RISC and have also determined the assembly mechanism [17–21]. The identification of R2D2 protein in* Drosophila*, RDE4 protein in* C. elegans*, and TRBP protein in humans [21–23] provided additional information about the molecular composition of RISC, as well as about the initiation and effector phases of the RNA interference mechanism. This evidence indicates that RISC activity is important during the initiation phase to generate microRNAs and suggests that RISC may be involved in later events of the RNA interference mechanism, that is, in the effector phase of posttranscriptional regulation of gene silencing. In the next stage of biogenesis, pre-microRNA processing and RISC assembly are functionally connected to catalyze multiple cycles of hydrolysis of specific target mRNA [15]. MicroRNAs 19–25 nucleotides in length are processed to produce mature microRNAs, which associate with RISC in order to recognize complementary mRNA sequences. MicroRNAs mediate their effects at the mRNA level by inhibiting translation or inducing cleavage of target mRNA, recognized by nucleotide complementarity between the microRNAs and mRNA. Perfect complementarity induces cleavage of mRNA, whereas partial complementarity with several mismatches leads to translation arrest [24]. Taken together, the evidence supports the idea that the RNA interference mechanism is a natural process of sequence-specific posttranscriptional gene silencing mediated by microRNAs in eukaryotic cells.

## 3. Disruption of MicroRNA Expression Profiles in Cervical Cancer

By comparing microRNA expression profiles between normal tissue and tumor tissue, studies have identified deregulated microRNAs and mRNAs, demonstrating an aberrant microRNA expression pattern in various malignancies [25]. Malignant changes such as tumorigenesis or tumor progression are associated with changes in the expression of multiple microRNAs, rather than a single microRNA regulating an oncogene or tumor suppressor target gene [26]. However, it is unclear whether microRNA expression is altered at the onset of cell transformation or as a consequence. Since microRNAs regulate the expression of their mRNA targets, it is expected that the over- or underexpression of microRNAs would have an effect on cellular phenotype [27]. For cancer research purposes, microRNAs can be divided in those with increased expression which target tumor suppressor protein and are known as oncomirs and those with decreased expression which generally target oncogenes and are referred to as tumor suppressor microRNAs. However, sometimes microRNAs overexpressed in cancer cells may act as tumor suppressors if they target oncogenes, and similarly microRNAs underexpressed in tumor cells sometime act as oncomirs if they target tumor suppressor genes. These classes of microRNAs have become an attractive target for gene therapy in recent years.

Several microRNAs with altered expression in cervical cancer have been identified and put forth as oncomirs or tumor suppressor genes. For instance, miR-10a, miR-106b, miR-21, miR-135b, miR-141, miR146, miR-148a, miR-214, and miR-886-5p have been proposed to act as oncomirs in cervical cancer, contributing to the development of cancer through dysregulation of gene products involved in cell proliferation, apoptosis, or cell-cell adhesion [3, 5, 28–33]. The miR-10a is an oncomir found to be overexpressed in colon and pancreatic cancer [34] which functions in tumor invasion and metastasis. A study by Long et al. [28] found miR-10a to be overexpressed in 92.8% of cervical cancer tissues, with similar results reported by Volinia et al. [34]. In the Long et al. study, researchers found an inverse correlation in the expression of miR-10a and of Close Homolog of L1 (CHL1) transmembrane protein type 1, a protein involved in cell adhesion, in HeLa (HPV18+) and C33 A (HPV−) cells transfected with pri-miR-10a. In these cells a decrease in CHL1 mRNA levels was observed, suggesting that miR-10a targets CHL1. Long et al. propose that a decrease in CHL1 caused by miR-10a interference results in deregulation of the MAPK and PAK pathways which affect downstream molecules, contributing to cell growth, migration, and invasion of cells into other tissues. These findings suggest that, in cervical cancer, miR-10a may play an important role in tumor metastasis by regulating the cellular gene CHL1 [28].

The expression of miR-21 has been found to be altered in almost all types of cancer and this microRNA has been classified as an oncomir. miR-21 has been reported to be overexpressed in glioblastoma, uterine leiomyosarcoma, diffuse large B-cell lymphoma, breast, lung, stomach, prostate, colon, esophagus, head and neck cancer, and cervical cancer [35]. miR-21 is located on chromosome 17 at the 17q23.2 locus and the gene coding for pri-miR-21 is located within the intronic region of the protein-coding gene TMEM49, a human homolog of rat vacuole membrane protein [35]. Inhibition of miR-21 can induce cell cycle arrest and increase chemosensitivity to anticancer agents, offering evidence that miR-21 may function as an oncogene in human cancer [36–38]. miR-21 has been shown to negatively regulate the expression of cellular genes p53 and Cdc25, which are involved in regulation of cell proliferation, RECK and TPM1, which suppress metastasis, and PDCD4 and PTEN which induce apoptosis of malignant cells. Furthermore, the repression of PDCD4 by miR-21 has been proposed to generate feedback signaling or autoregulation by activating mitogenic signals throughout the RAS pathway, which leads to the induction of the AP-1 transcription factor. AP-1 may bind to specific recognition sites into the miR-21 promoter region to activate its transcription [35]. This evidence implicates miR-21 in multiple malignancy-related processes in cervical cancer including cell proliferation, apoptosis, invasion, and metastasis.

Another microRNA overexpressed in cervical cancer is miR-886-5p. Li et al. showed that miR-886-5p negatively regulates the expression of Bax. [33]. Bcl-2 and related proteins such as Bcl-XL increase cell survival, and their functions are countered by related proapoptotic proteins such as Bax and Bak. Specifically, the Bax gene codes for a proapoptotic protein that is inserted into the outer mitochondrial membrane in response to a cell death signal, causing release of cytochrome C and subsequent activation of the initiator caspase-9, resulting in apoptosis. Bax expression levels are reduced in cervical cancer cells. In HPV16+ H8 cells, low levels of Bax have been associated with decreased apoptosis and increased cell proliferation, and silencing miR-886-5p in HPV16+ SiHa cells increases levels of Bax and apoptosis. These data suggest that miR-886-5p regulates Bax expression via translational inhibition and this regulatory pathway may play an important role in cancer cervical development.

Some microRNAs act as tumor suppressor microRNAs which regulate oncogenes and are underexpressed in cervical cancer; examples of these include let-7c, miR-124, miR-126, miR-143, and miR-145 [3]. One of the first microRNAs studied was let-7.  The 3′-UTR region of the human RAS gene contains multiple let-7 complementary sites (LCSs), enabling let-7 to regulate Ras expression [39]. RAS proteins are membrane proteins that regulate normal cell growth and differentiation throughout NF-kB transcription factor, PKB/Akt and MAPK kinases. In lung cancer, let-7 is underexpressed relative to normal tissue, while levels of RAS are significantly higher than in normal tissue, which is consistent with a potential let-7-mediated mechanism in cancer development. With regard to cervical cancer, let-7 is expressed at a frequency of 15% to 18% in individual normal cervical samples while its expression was not detected in three cancer cell lines (SW756, C4I, and SiHa cells) and detected at 0.2% in CasKi cells (HPV16+), 0.4% in ME-180 cells, and 7.2% in C33 A cells [40]. In HeLa cells expressing endogenous let-7 that were transfected with antisense oligonucleotides designed to inhibit let-7 activity, a ~70% increase in RAS protein levels was observed, strongly suggesting that let-7 negatively regulates the expression of RAS in human cervical cells [39].

Another important tumor suppressor microRNA in cervical cancer is miR-143. The genes targeted by miR-143 include k-Ras, Macc1, and Bcl-2, which are implicated in the ERK5 and MAPK signaling pathways [41, 42]. One of the target genes, Bcl-2, is an oncogene which acts to suppress cellular apoptosis; therefore, overexpression of Bcl-2 inhibits apoptosis in damaged cells, leading to uncontrolled cellular proliferation that drives the development of cancer. In addition, overexpression of the BCL-2 protein may contribute to metastasis in certain cancers. Liu et al. demonstrated that overexpression of miR-143 in HeLa cells resulted in suppression of Bcl-2, while knockdown of miR-143 increased Bcl-2 expression. Overexpression of miR-143 induced with anti-miR-Bcl-2 partially reversed the inhibition of cell proliferation and promoted apoptosis in the HeLa cells expressing miR-143 [43]. Another experimentally verified target of tumor suppressor microRNAs miR-143 and miR-145 is the cellular gene ERK5 (also known as MAPK7) a mitogen-activated protein kinase (MAPK) regulated by a wide range of mitogens and by cell stress, which promotes cell growth and proliferation in response to tyrosine kinase signaling [44]. In the bladder cancer cell line T24, prostate cancer cell lines LNCaP and C4-2, and the Burkitt lymphoma cell line Raji, ERK5 expression levels were found to be reduced and cell proliferation was inhibited in response to increased levels of miR-143 and miR-145 [40, 45–47]. Study of the role of ERK5 in cervical cancer and its regulation by miR-143 y miR-145 is certainly worthy of further investigation. Recently, Zhang et al. demonstrated that IFN-*β* is induced by miR-129-5p in HeLa cells (HPV18+) [48]. They identified that miR-129-5p overexpression inhibits the growth of HeLa cells and that transfection of pre-miR-129-5p increased the arrest of HeLa cells and decreased the HPV18 E6 and E7 expression. The same group observed that miR-129-5p expression was induced by INF-*β*. In addition, they demonstrated that the SP1 transcription factor can be downregulated by overexpression of miR-129-5p throughout a binding site for miR-129-5p at SP1 3′-UTR. These data support the notion that induced expression of miR-129-5p by IFN-*β* suppresses the progression of cervical cancer cells by downregulating HPV18 E5 and E7 expression, and SP1 transcription factor is direct downstream, target of miR-129-5p.

## 4. Cell Checkpoints and Regulation of MicroRNAs in Cervical Cancer

A significant event in HPV-associated carcinogenesis is the induction of genetic instability and global disruption of cell gene expression principally by the HPV E6 and E7 oncoproteins, whose cell protein targets have been identified via molecular analysis. One of the main, well-defined targets of HPV E6 is the tumor suppressor protein p53 which acts as a checkpoint to maintain cell homeostasis. The p53 transcription factor is the tumor suppressor gene most frequently inactivated in human cancers and is involved in the control of cell proliferation and the response to genotoxic stress and DNA damage [49]. The inactivation of p53 by E6 affects multiple cell processes including apoptosis, cell cycle arrest, cellular differentiation, and senescence [50]. Thus, loss of p53 results in an increase in the genomic instability of the cell. On the other hand, the HPV E7 oncoprotein may interact with the pRb tumor suppressor family of proteins, another important checkpoint, to liberate transcription factors in the E2F family, thus stimulating the expression of multiple genes involved in cell cycle progression [50, 51]. In addition to their oncogenic and antiapoptotic effects, the E6 and E7 oncoproteins may modulate viral transcription and other cellular genes. Thus, HPV E6 and E7 have effects on several levels of cellular functions, such as cell cycle control and regulation of gene expression, and in combination they efficiently immortalize and transform human keratinocytes to promote carcinogenesis. Due to these properties, HPV E6 and E7 oncogenes are an important focus of research to improve understanding of the molecular mechanism of viral oncogenesis in humans and are considered good targets for gene therapy for cervical cancer.

Several mechanisms likely contribute to the global deregulation of microRNAs which has been reported in cervical cancer cells. For instance, there is growing evidence that microRNAs are critical components of several canonical and noncanonical signaling pathways that frequently undergo gain or loss of function in tumor cells, including those regulated by key molecules to maintain cell homeostasis. One such pathway involves the c-Myc gene, which encodes a helix-loop-helix transcription factor with several functions including inducing cell cycle progression and which represents a checkpoint in cell homeostasis. As such, c-Myc is one of the most frequently activated oncogenes in human cancers. In cervical cancer, independent of HPV E6 p53-degradative function, E6 interacts with c-Myc to enhance c-Myc binding to the hTERT promoter and induce hTERT expression [52]. Veldman et al. demonstrated that E6/c-Myc/hTERT regulation is mediated by the proximal Myc/Max-binding element (E-box) in the hTERT promoter, which is the main determinant of E6 and c-Myc responsiveness. Furthermore, c-Myc represses transcription when it binds to promoters by transcriptional activators such as Miz-1 (Myc-interacting zinc-finger protein 1), a transcription factor involved in transcriptional activation of p15 and p21 genes. In cervical cancer cells E7 has been shown to form a complex with Miz-1 which has effects on p21 regulation as well as on cell cycle progression in response to UV-induced DNA damage [53]. In addition to c-Myc directly controlling the expression of many protein-coding genes, there is an increased appreciation that its ability to reprogram microRNAs expression also contributes to its oncogenic process. O'Donnell et al. reported that the c-Myc oncogene directly regulates microRNA expression [54]. Moreover, Ota et al. demonstrated that miR-17-92 cluster is transactivated by c-Myc [55]. They performed ChIP assay which demonstrated that c-Myc interacts directly with a conserved binding site in the first intron of miR-17-92 primary transcript to activate its transcription. Other studies of microRNA regulation by c-Myc have revealed a wide role for this transcription factor in reprogramming microRNA expression. For example, Chang et al. demonstrated that c-Myc activation leads not only to induction of the miR-17-92 cluster but also to widespread repression of microRNAs including let-7 family, miR-29 family, miR-15a/16-1, and miR-34a which are known to have antitumorigenic activity [56]. Through ChIP assays they demonstrated that c-Myc associates with the core promoter of the microRNAs it represses, suggesting that this microRNA downregulation is a consequence of reduced transcription of pri-microRNAs. This evidence indicates that c-Myc overexpression inhibits the expression of several microRNAs such as let-7a-1/f-1/d, miR-15a/16-1, miR-22, miR-26a-2, miR-26b, miR29a/b-1, miR-29b-2/c, miR-50e/30c-1, miR-34a, and miR-146a, which may be the result of c-Myc binding to microRNA promoters.

Numerous downstream targets of the microRNA-17-92 cluster have been identified, providing evidence about the oncogenic mechanisms mediated by this microRNA. Downregulation of the proapoptotic genes (CL2L11/BIM) by multiple members of the microRNA-17-92 cluster likely explains these microRNAs' ability to block apoptosis and promote carcinogenesis processes [57]. MiR-20a is a member of the miR-17-92 cluster, and its function in cervical cancer cells is not clear. Kang et al. confirmed that miR-20a is upregulated in cervical cancer tissue [58]. Overexpression of miR-20a in the cervical cancer cell lines HeLa and C33A increased cell proliferation, migration, and invasion, whereas inhibition of miR-20a suppressed those functions. This same group found that the oncogene TNKS2 is directly upregulated by miR-20a, with effects on cervical cancer cell colony formation, migration, and invasion.

The main reported function of HPV E6 is to target p53 for degradation. Thus, it is certainly conceivable that HPV E6 is able to regulate the expression of many cellular microRNAs through p53, which acts as a checkpoint to maintain cell homeostasis. The function of p53 is critical and more complex than originally thought, because it represses and/or activates coding and noncoding genes during their biogenesis and at the level of transcription. p53 binds to several microRNA promoters to induce repression. p53-mediated repression of miR-17-5p, miR-18a, miR-18b, miR-19a, miR-19b-1, miR-19-2, miR-20a, miR-20b, miR-25, miR-92-1, miR-92-2 miR-93, miR-106a, and miR-106b has been reported [59]. HPV E6-expressing cells show a p53 null phenotype; consequently, all microRNAs regulated by p53 are likely affected by E6. Wang et al. had reported that miR-34a is regulated by E6-dependent expression in cervical cancer [60]. They observed that cervical cancer tissues and cervical cancer-derived cell lines infected with high-risk HPVs display reduced expression of tumor suppressive miR-34a. The decrease in miR-34a expression correlates with the early productive phase and is attributed to the expression of HPV E6, which destabilizes p53, a known miR-34a transactivator. This same group demonstrated that knockdown of E6 in cervical cancer cell line (HPV16+ and HPV18+) by siRNAs leads to an increased expression of p53 and miR-34a and accumulation of miR-34a in G0/G1 phase cells. Furthermore, they demonstrated that miR-34a gene is a direct transcriptional target of p53 and its expression can be transactivated by the binding of p53 to a consensus p53 recognition site in the miR-34a promoter region. Finally, they found that p53 degradation by E6 leads to the decreased miR-34a in raft cultures, CIN, and cervical cancer tissues [60]. Given that miR-34a has effects on the expression of many cell cycle regulators, including cyclin E2, cyclin D1, CDK4, CDK6, E2F1, E2F3, E2F5, Bcl-2, SIRT1, and p18, the repression of p53 and miR-34a disrupts the multistep control of cell cycle progression, senescence, and apoptosis, resulting in disruption of cell differentiation and proliferation and leading to cell transformation [61].

**Figure 1:**
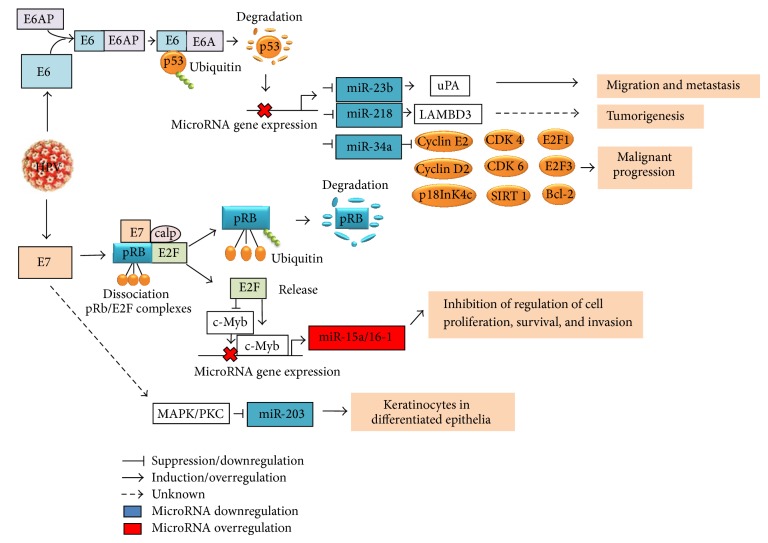
Schematic model of the interaction between microRNAs and factors involved in malignant transformation caused by HPV E6 and E7 expression in cervical cancer cell. Cervical cancer is the second most common cause of cancer mortality in women worldwide and persistent infection with HPV is the main etiologic agent. HPV E6 and E7 deregulate cellular proliferation and inhibit the apoptotic mechanism by targeting p53 and pRb, respectively. In addition, E6 disrupts the expression of miR-23b, miR-218, and miR-34a via p53 degradation and their expression is transactivated by the binding of p53 to consensus sites in the promoter regions, affecting the expression of cell cycle regulators, such as E2, cyclin D1, CDK4, CDK6, E2F1, E2F3, E2F5, Bcl-2, SIRT1, p18, uPA, and LAMBD3. In the overexpression of miR-15/16 cluster by E7, E2F1 transactivates the c-Myb expression and represses the c-Myc expression, and then the microRNA cluster regulation is controlled by binding of c-Myc or c-Myb to promoter region of microRNA cluster. The increased expression of miR-15a/miR-16-1 induces the inhibition of cell proliferation, survival, and invasion. The downregulation of miR-203 by E7 is mediated by MAPK/PKC pathway.

The tumor suppressor microRNAs miR-15a and miR-16-1 are expressed as a microRNA cluster from an intron region of the DLEU2 (Deleted in Lymphocytic Leukemia 2) transcript and influence cell proliferation, survival, and invasion. Wang et al. demonstrated higher levels of miR-15a and miR16-1 expression in cervical cancer tissues compared to normal cervical tissues; however, the overexpression of this microRNA cluster does not appear to affect growth of cervical cancer cells [3]. To analyze whether miR-15a/miR-16-1 cluster overexpression is associated with high-risk HPV infection, Zheng and Wang compared miR16-1 expression levels in raft tissues derived from human foreskin keratinocytes with and without HPV18 infection and observed a twofold increase in miR-16-1 expression in the HPV18+ raft tissues [62]. When cells were infected with a retrovirus expressing HPV18 E6 and E7, expression of miR-16-1 was increased only in the raft tissues expressing HPV E7, but not in those expressing HPV E6, suggesting that while HPV oncoproteins regulate expression of the miR-15/16 cluster, E7 is specifically responsible for the increased expression of miR-16-1. This evidence indicates that these cell checkpoints can be studied to improve our understanding of the gene expression network in the context of biological systems.  Figure 1 summarizes the main checkpoints and the pathways in which microRNAs are involved in cervical cancer cells.

## 5. Regulatory Genetic Networks Modulated by MicroRNAs in Cervical Cancer

Recently, Wang et al. described regulatory genetic networks in cervical cancer, which were constructed from databases of differentially expressed microRNA and related genes [59]. Three regulatory genetic networks were generated; the first was the differentially expressed network which includes differentially expressed genes, differentially expressed microRNAs, and host genes of differentially expressed microRNAs. The second network was constructed from related genes, related microRNAs, and host genes of cervical cancer-related microRNAs. The third network was the global network, which consists of all the elements extracted from the basic source data. In the first network, seven genes and ten microRNAs were experimentally validated as differentially expressed in cervical cancer. The subnetwork centered around PTEN was the principal component. TWIST1, miR-214, PTEN, and miR-21 function together as an ordered chain of control. The miR-21 targets its regulator PTEN, which formed a self-adapting feedback loop, creating a balance mechanism with STAT3 and miR-21 in the system as components. Thus, two host genes, two transcription factors, and one microRNA influence the expression of PTEN and miR-21. The circling between miR-21 and PTEN makes them dominant and dominating factor simultaneously and turns the pathway into a bidirectional net crux. In another partial network centered on miR-143, TP53 and TGF*β*1 jointly have been shown to regulate miR-143. The second network expands on the differentially expressed network by including more genes and microRNAs whose association with cervical cancer is not as close as the differentially expressed ones. The related network is of a much larger scale and of higher complexity than the differentially expressed network. Certain factors are predominant, including microRNAs such as miR-21, miR-23b, miR-34, miR-143, and let-7c and transcription factors of PTEN, TP53, TP63, c-Myc, and k-Ras. Two more self-adapting feedback mechanisms were identified involving let-7c, miR-34, and c-Myc. c-Myc acts as transcription factor for let-7b and let-7c, which target k-Ras. This is accorded to inhibition of the transactivation function of c-Myc. In addition, c-Myc's overexpression could alter the suppressive function of let-7c. Meanwhile, another tumor suppressor, miR-34, has a local balance adjustment system with c-Myc as well. In the third network, interactions involving the previously identified transcription factors and their base sequences of 1000-nt were evaluated; these transcription factors were integrated with host genes and differentially expressed microRNAs to include 1000-nt transcription factors in the network system of cervical cancer. Self-adapting feedback was assessed and was found to exist between NF-kB and miR-21. NF-kB regulates miR-21, miR-214, and let-7b. MiR-21 and miR-214 are core biological factors in the cervical cancer network and their appearance here supports the importance of these particular microRNAs. With the help of miR-21 and mir-214, NF-kB participates in the subsystem centered around PTEN and miR-21. This method of analysis could be useful to identify more core factors, other parallel networks and relevant motifs in cervical cancer carcinogenesis, and/or other tumorigenesis processes.

## 6. The MicroRNA Profile Expression in the Natural History of Cervical Cancer

Cervical cancer is histologically classified into squamous cell carcinomas (SCCs), which comprise about 80% of cervical cancers, adenocarcinomas (AdCAs), which comprise about 15%, and adenosquamous carcinomas, which comprise about 3% to 5%. According to the Bethesda classification, preneoplastic cell abnormalities that precede cervical cancer are divided into low-grade intraepithelial lesions (cervical intraepithelial neoplasia (CIN-1)) and high-grade lesions (CIN2-3). A number of studies have demonstrated deregulated patterns of microRNA expression in cell lines derived from cervical cancer tissue as well as in tissue samples; however, only a few have described the alteration of microRNA expression that occurs during the progression from normal cervical epithelium to high-grade CIN lesions to SCC or AdCAs.  Table 1 summarizes the existing evidence in this area.

Wilting et al. determined that the expression profiles of microRNA in CIN2-3 are similar to normal epithelium infected with high-risk HPV, consistent with the concept that cervical lesions take approximately a decade to progress to invasive cervical carcinoma [6]. The authors identified 106 microRNAs that are differentially expressed (*P* < 0.01) in CIN2-3 and/or SCCs compared to normal epithelium. One set of 27 of these microRNAs showed differential expression (false discovery rate FDR < 0.05) only in CIN2-3 compared to normal tissue and did not have differential expression in SCC compared to normal tissues (FDR > 0.1). These microRNAs were therefore designated microRNAs with transient early expression. A different set of 33 microRNAs showed concordant differential expression (one FDR < 0.05 and the other FDR < 0.1) in both CIN2-3 and SCC and were thus designated microRNAs with continuous early expression. A third set of 46 microRNAs showed differential expression (FDR < 0.05) restricted to SCC and were thus classified as microRNAs with late expression. These 46 microRNAs are potentially important in the progression of high-grade CIN lesions to invasive carcinomas. Finally, one set of 18 microRNAs showed significantly different expression between AdCA and SCC and could be used to accurately diagnose the different histological types of cervical cancer.

To characterize changes in microRNA expression associated with CIN progression, Pereira et al. analyzed the microRNA expression profile in CIN-1, CIN-3, and cervical cancer and identified 21 microRNAs with statistically significant differential expression between CIN-1 and/or CIN-3 and the pool of normal samples (*P* < 0.05) [4]. One set of eight microRNAs exhibited relative decreased expression in the transition from normal cervix to CIN1-3 and from CIN1-3 to cancer. Another set of six microRNAs displayed relative decreased expression in the transition from normal cervix to CIN1/3 but increased expression from CIN1-3 to cervical cancer. Two microRNAs exhibited relative increased expression in the transition from normal cervix to CIN1-3 and decreased expression from CIN1-3 to cervical carcinoma. Interestingly, one set of five microRNAs displayed relative increased expression in the transition from normal cervix to CIN1/3 and from CIN1/3 to cancer. In another study, Li et al. compared HPV16+ SCC, HPV16+ CIN2-3, and normal cervical tissue and identified a set of 31 unique microRNAs with significant and continuous expression trends in the progression from normal tissue to cancer, with 14 microRNAs decreasing in expression and 17 microRNAs increasing in expression [5]. Among these, miR-218 was the most significantly downregulated, with log_2_ values of −1.15-fold and −4.83-fold changes in CIN2-3 and SCC, respectively. These data were validated with increased sample number for each HPV16+ histological type, which confirmed an identical microRNA expression profile to the primary screening. Lui et al. analyzed microRNA expression levels in human cell lines transformed with the HPV16/18 as well as in normal cervical cells and determined that miR-21 is overexpressed in HPV16/18+ cervical cancer cell lines derived at a rate of 45% compared to 13% expression in normal cells, miR-143 is not expressed in HPV+ cells, and let-7 and miR-196 are underexpressed in HPV-transformed cells compared to normal cervical cells [40]. In summary, this evidence supports the theory that microRNAs have specific expression profiles at different stages in the natural history of cervical cancer associated with high-risk HPV infection. The variation in the microRNA expression pattern is associated primarily with HPV E6 and E7 oncoprotein expression. By comparing the microRNA expression profile in CIN1-2-3 and cancer to normal tissue, it is possible to establish a specific genomic and/or transcriptomic signature of cervical tissue infected with HPV. Thus, microRNA expression profiles may be employed as biomarkers in the staging and prognosis of cervical cancer associated with high-risk HPV infection.

## 7. siRNAs for HPV Oncogenes as Potential Gene Therapy for Cervical Cancer

The silencing of genes by siRNAs is a potential mechanism to inactivate foreign DNA sequences and may be employed to silence the expression of HPV oncogenes in cervical cancer. The first studies carried out with synthetic siRNAs to silence HPV16 E6 and E7 oncogene expression were described by Jiang and Milner in 2002 [63]. In this study, the administration of siRNAs led to mRNA cleavage and the specific silencing of HPV16 E6 and E7 oncogene expression. Furthermore, E6 silencing induced expression of the p53 gene and transactivation of the p21 gene and decreased cell proliferation, while silencing of E7 induced cell death by apoptosis. These findings demonstrated for the first time that the expression of HPV E6 and E7 oncogenes may be specifically silenced by siRNAs in human tumor cervical cells HPV+.

Recent investigation has focused on silencing the HPV E6-E7 bicistron with siRNAs. These oncogenes are transcribed jointly, as a bicistron, which is the result of alternative splicing. The effect of silencing of both E6 and E7 with synthetic siRNAs against the HPV16 E6 oncogene in SiHa cells (HPV16+) has been described [64]. The data demonstrate inhibition of cell proliferation, p53 and pRb protein expression, and p21 induction. The effect of siRNAs against the HPV16 E6 oncogene on the E6-E7 bicistron has been studied* in vitro* as well as* in vivo*. Administration of siRNAs for E7 induces silencing of both oncogenes, while siRNAs for E6 inhibit E6 expression but do not affect E7 expression [65, 66]. These same studies analyzed the functionality of siRNAs for E6 and E7 and demonstrated induction of expression of p53, p16, p21, p27, and pRb, silencing of cyclin A gene, and induction of apoptosis in cervical cancer cells. Another group has reported the use of synthetic siRNAs to silence the HPV18 E6 oncogene [67]. This study demonstrated induction of apoptosis of CasKi cells (HPV16+), increased p53 and p21 expression, and expression of pRb. However, the siRNAs for HPV18 E6 did not affect HPV18 E7 expression. Sima et al. generated siRNA for HPV16 E7 in the pSIRE-DNR plasmid for the generation of small transcripts [68]. Their group demonstrated silencing of the HPV16 E6-E7 bicistron in SiHa and CaSki cells (HPV16+), with a resultant increase in expression of p53, p21, and pRb and induction of cervical tumor cell death by apoptosis. This evidence supports the idea that the silencing of HPV E6-E7 bicistron expression is dependent on the design of the siRNA sequences and suggests that the alternative splicing of HPV E6 and E7 oncogenes precedes the silencing by siRNAs.

## 8. Chemotherapeutic Drugs and siRNAs for HPV E6 and E7

Although the effect of chemotherapeutic drugs on p53 expression in cervical cancer cells is known, new research has focused on the association between the activation of p53 gene, the cytotoxic effect of drugs, and the silencing of HPV oncogenes with siRNAs. Different groups have analyzed the expression of p53 in HeLa cells (HPV18+) treated with siRNAs for HPV18 E6, combined with carboplatin, cisplatin, doxorubicin, etoposide, gemcitabine, mitomicine, mitoxantrone, oxaliplatin, paclitaxel, and/or topotecan treatment [69]. The researchers observed silencing of HPV18 E6 and E7 oncogenes, as well as an increase in p53 protein expression and changes in cytotoxicity dependent on the nature of each chemotherapeutical compound. A separate group found that the administration of siRNAs for HPV18 E6 generated in lentivirus, combined with cisplatin, in HeLa cells produced silencing of HPV18 E6 and E7 oncogenes, an increase in p53 expression, and death of cancer cells by cellular senescence [70]. This evidence suggests that the silencing of HPV E6 and E7 oncogenes with siRNAs can increase cellular sensitivity to the cytotoxic effects of drugs and that combined treatment may have a synergistic effect and reduce resistance to chemotherapeutical drugs, representing an advantage for treatment.

## 9. Transport Vehicles for siRNAs

When siRNAs are administered via lipofection to mammalian cells, one potential problem is cleavage of siRNAs by endogenous cellular endonucleases. An alternative technique to protect siRNAs from this cleavage is the synthesis of siRNAs with chemical modifications; however, this may induce undesirable collateral effects. Another problem that arises in the systemic administration of siRNAs is that there is no dose-dependent effect on target organs. In order to overcome these methodological complications, siRNAs for HPV oncogenes may be administered via a liposome-based system contained in biogels, which has been shown to cause specific silencing of E7 and induction of apoptosis of cancerous cells* in vitro* [71]. It has been demonstrated that when siRNAs are administered in CasKi cells (HPV16+), silencing of both oncogenes occurs and the cells die by apoptosis. The effects of siRNAs for E6 were evaluated in a murine tumor model, with resultant silencing of the viral oncogene as well as induction of tumor cell apoptosis and significant inhibition of the growth of the tumor mass* in vivo* [72]. In addition, atelocollagen has also been used as vehicle to administer siRNAs for HPV18 E6 and E7, with demonstration of the silencing of E6 and E7 oncogene expression, inhibition of cell proliferation, induction of the expression of pRb, and death of cancer cells by cellular senescence [73].

Although the silencing effects of siRNAs are evident, the half-life of these molecules after administration is relatively short, even when they are attached to transport molecules, which limits their application in preclinical or clinical trials. Furthermore, application of siRNA for HPV oncogenes in clinical studies requires development of highly specific siRNAs and more efficient systems for* in vivo* release. To this end, protocols have been developed to use lentiviruses as molecular vectors for siRNAs, as well as for the stable transfection and transduction of siRNAs in human cervical cancer cells [74]. The use of plasmids to clone siRNAs affords a longer half-life and greater stability to these molecules, and the most efficient molecular vehicles to release these plasmids are the lentiviruses. The lentiviruses have advantages such as the ability to infect dividing cells and resting cells with great efficiency, being nontoxic and only mildly immunogenic, and have been modified not to integrate themselves into the cellular genome. Their application has been studied in preclinical and clinical trials with encouraging results. In summary, siRNAs for HPV oncogenes that are generated in lentivirus have direct application in tumor sites and have been proven to be comparatively more useful than synthetic siRNAs.

## 10. Design of siRNA Sequences for HPV E6 and E7

A large volume of software has been developed and is available at diverse websites which allows for the design of more efficient and biologically functional siRNA sequences. For example, siDirect software has been used for the design of siRNA sequences that are highly efficient, with high specificity for the target gene sequence [75]. The siDirect software minimizes nonspecific complementarity of siRNA sequences to reduce binding to unrelated sequences. To do this, siDirect software uses a rigorous specificity measure called mismatch tolerance which involves identifying the minimal number of nonspecific sequences among the siRNA sequences and any sequence that does not correspond to the target sequence. Highly efficient siRNA sequences are selected from the algorithm based on the rules developed by Ui-Tei et al. [75, 76]. The siDirect software has been utilized to generate a set of several siRNAs for HPV16 E6 and E7 oncogenes [76, 77]. To evaluate the functionality of these siRNAs, the DNA inserts were cloned in the silencing plasmid psiCheck2. Highly specific silencing of E6 and E7 was demonstrated, along with suppression of the proliferation of HPV 16+ tumor cervical cells, an increase in the expression of p53 and p21, and morphologic and histochemical changes characteristic of cancer cell death by cell senescence. The functionality of siRNAs was also demonstrated* in vivo*, producing inhibition of tumor growth in an HPV16+ animal tumor model. Taken together, this evidence demonstrates that software-generated siRNAs are functional* in vitro* as well as* in vivo* and confirms that siRNAs can silence expression of HPV E6 and E7 oncogenes, with biological effects on cervical cancer cells.

Our group has also developed a protocol to silence the expression of HPV16 E6 and E7 oncogenes using siRNAs [78]. In this study, we designed siRNAs for HPV16 E6 and E7 with siRNA Target Finder software. The siRNAs for HPV16 E6 and E7 were designed for cloning into the pSilencer 1.0-U6 plasmid. The silencing of HPV16 E6 and E7 oncogenes and the biological effect of siRNAs on SiHa cells (HPV16+) were evaluated. SiHa cells showed a selective decreased expression of mRNA HPV16 E6 and E7 oncogenes and oncoproteins, as well as functional effects in cell proliferation, an increase in p53 and pRb protein expression, and features of autophagy and apoptosis, attributable to silencing of E6 and E7 oncogenes. In a murine tumor model, the administration of siRNAs reduced tumor growth rate. These findings suggest that selective silencing of HPV16 E6 and E7 oncogenes by siRNAs has significant biological effects on survival of human cancer cells* in vitro* and* in vivo* and represents a potential gene therapy against cervical cancer.

## 11. Transcriptome Regulation by siRNAs for HPV E6 and E7

Studies have examined the effect of silencing HPV E6 and E7 oncogenes using siRNAs on transcriptome regulation in human cervical cancer cells. Kuner et al. analyzed the transcriptome of HeLa cells and patient biopsies after silencing HPV 18 E6 and E7 with siRNAs generated in the silencing plasmid pSUPER [79]. The study identified 360 cellular genes which were negatively regulated and 288 genes which were positively regulated due to silencing of E6 and E7. Most of these genes are involved in biological processes that occur during development of the tumor cell, such as apoptosis control, cell cycle regulation, formation of the mitotic spindle, processing of mRNA by splicing, metabolism, DNA replication and repair, nuclear transport, cell proliferation, and gene regulation by c-Myc. These findings complement previous studies which analyzed expression of the HPV E2 protein. E2 inhibits HPV E6 and E7 expression and alters the transcriptome expression in human tumor cervical cells. The potential of this type of studies lies in the fact that the basic cell pathways for viral transformation may be identified, which may be targets for the development of therapeutically strategies. With this strategy, new molecular biomarkers may be identified for diagnosis and prognosis of cervical cancer.

## 12. Conclusions and Perspectives

Review of the evidence regarding microRNA expression in cervical cancer reveals that the expression pattern of microRNAs in cancer cell lines and cervical (pre)malignant lesions provides valuable information about the role of microRNAs in the different stages of cervical carcinogenesis. The microRNAs may represent a promising disease biomarker in cervical cancer, as well as potential therapeutic targets in gene therapy. The microRNAs regulate a large number of target genes involved in cell proliferation, differentiation, and apoptosis, and there is evidence* in vitro* and* in vivo* of their function as oncogenes or tumor suppressors in cervical cancer. The transcriptional regulation of microRNAs via epigenetic mechanisms or transcription factors must be further studied to understand their role in the process of carcinogenesis. Other important aspects to consider are chromosomal damage that may lead to overexpression or downregulation of microRNAs and the genomic localization of microRNAs in fragile sites that may undergo amplification or deletions near HPV integration sites in cervical cancer. Additionally, microRNAs may enhance the current diagnostic technologies in cervical cancer. Identification of key microRNAs and cellular target genes involved in HPV-related pathways based on expression patterns in HPV-infected cervical tissues provides useful information about prognosis. The identification and subsequent functional evaluation of host microRNA expression profiles associated with HPV oncoproteins is the major challenge in utilizing microRNAs as molecular biomarkers, and better understanding of their role in cervical carcinogenesis may allow for the development of specific targeted strategies against cervical cancer. In regard to the function of siRNAs, the evidence supports the idea that administration of siRNAs for HPV E6 and E7 oncogenes induces silencing of viral oncogene expression. The administration of siRNAs has biological effects on human tumor cervical cells transformed by HPV, including activation of cell death by apoptosis, effects on cell senescence, synergistic cytotoxicity with drugs used in chemotherapy for cervical cancer, and inhibition of cancer cells' tumor growth potential* in vivo*. The relevance of the silencing properties of the E6 and E7 oncogenes will be better appreciated once they are applied in clinical protocols. This will require adequate analysis during design of siRNA sequences to induce silencing of the E6-E7 bicistron, selection of optimal cloning vectors for siRNAs, and selection of siRNA transport vehicles such as biogels or atelocollagen to protect them from the action of endonucleases and to allow for administration in a site-specific and dose-dependent manner, as well as the development of treatment schemes which combine siRNAs, chemotherapeutic drugs, and/or radiation therapy. In summary, the use of siRNAs as a gene therapy strategy against cervical cancer has great potential for success in the treatment of this malignancy.

In regard to circulating microRNAs, Wang et al. demonstrated that serum microRNAs can be used as noninvasive biomarker for cervical SCC patients [80]. They show that miR-646, miR-141^*^, and miR-542-3p expression levels were significantly different between cervical SCC serum samples and the control samples of a total of 765 analyzed circulating microRNAs. They identified that miR-21, miR-200a, miR-143, miR-15a, miR-181c, miR-646, and miR-370 expression levels in serum can be used as biomarkers to monitor therapeutic efficiency. This evidence suggests that select microRNAs target genes were predicted to affect main biological processes such as hormone-mediated signal pathways and chemotherapy responses.

## Figures and Tables

**Table 1:** Differentially expressed profile microRNAs in normal squamous epithelium, CIN1-3, and CC.

MicroRNAs expression	Normal tissue	CIN1–CIN3	CC	Reference
miR-26a, miR-143, miR-145, miR-99a, miR-203, miR-513, miR-29a, miR-199a	Upregulated	Downregulated	Downregulated	Pereira et al. [4]
miR-106a, miR-205, miR-197, miR-16, miR-27a, miR-142-5p	Upregulated	Downregulated	Downregulated	
miR-522, miR-512-3p	Upregulated	Upregulated	Downregulated	
miR-148a, miR-302b, miR-10a, miR-196a, miR-132	Downregulated	Upregulated	Upregulated	

miR-155, miR-92a, miR-92b, miR-224, miR-221, miR-222, miR-31, miR-182, miR-106a, miR-17, miR-20a, miR-20b, miR-15b, miR-16, miR-25, miR-185, miR-93	Downregulated	Downregulated	Upregulated	Li et al. [5]
let-7b, miR-145, miR-126, miR-199a-3p, miR-195, miR-29a, miR-375, miR-10b, miR29c, miR-218, miR-424, miR-100, miR-125b, miR99a	Upregulated	Upregulated	Downregulated	

miR-192, miR-135b, miR-101, miR-191, miR-34c-5p, miR-150, miR-125a-5p, miR-30a, miR-143, miR-146b-5p, miR-181b, miR-7g, miR-26a, miR-29a, miR-29c, miR-29b, miR-10a, miR-29a, miR-145	Downregulated	Upregulated “early transient”	Upregulated “early transient”	Wilting et al. [6]
miR-205, miR-27a, miR-27b, miR-221, miR-193a-3p, miR-212, miR-770-5p, miR-484, miR-636	Upregulated	Downregulated “early transient”	Downregulated “early transient”	
miR-28-5p, miR-338-5p, miR-206, miR-200a, miR-92b, let-7i, miR-181d, miR-92a, miR-30e, miR-34b, miR-592, miR-19b, miR-106b, miR-595, miR-34a, miR-25, miR-146a, miR-21	Downregulated	Upregulated “early continuous”	Upregulated “early continuous”	
miR-203, miR-638, miR-370, miR-575, miR-193b, miR-149, miR-210, miR-622, miR-23b, miR-493, miR-296-5p, miR-671, miR-134, miR-365	Downregulated	Downregulated	Upregulated “early continuous”	
miR-30c, miR-425, miR-24, miR-331-3p, miR-151-3p, miR-107, miR-652, miR-17, miR-9, miR-185, miR-339-5p, miR-18a, let-7-d, miR-17, miR-30d, miR-130b, miR-15a, miR-106a, miR-19a, miR-200c, miR-20b, miR-363, miR-155, miR-141, miR-93, miR-15b, miR-16	Downregulated	Upregulated “late”	Upregulated “late”	
miR-125b, miR-375, miR-99a, miR-188-5p, miR-148a, miR-671-5p, miR-199b-3p, miR-513b, miR-378, miR-195, miR-486-5p, miR-26b, miR-376a, miR-199a-5p, miR-497, miR-100, miR-660, miR-218	Upregulated	Upregulated	Downregulated “late”	
